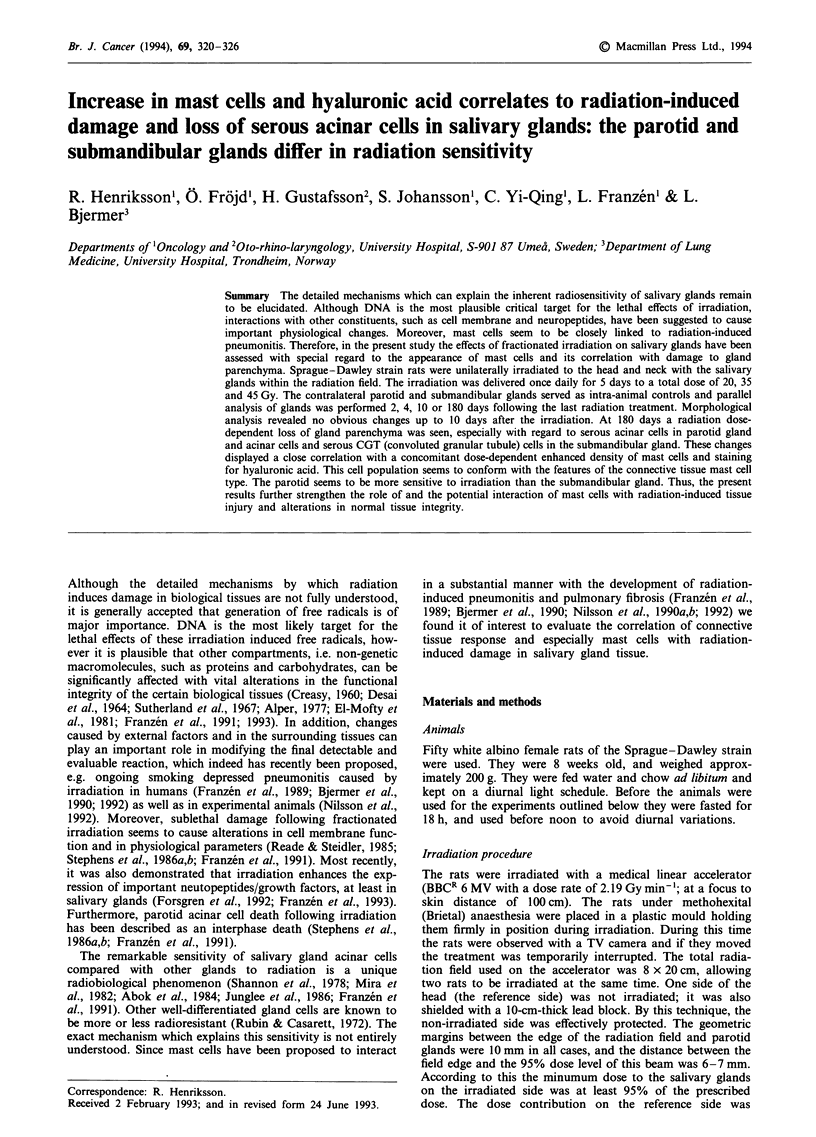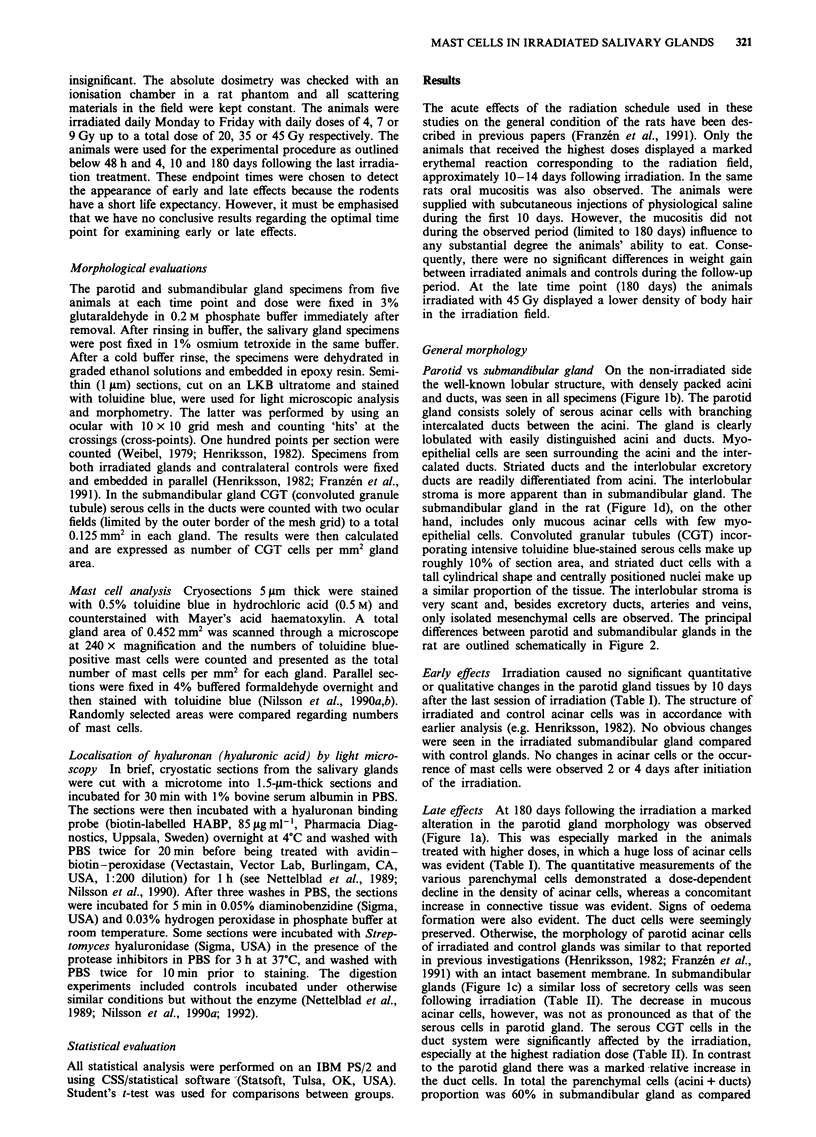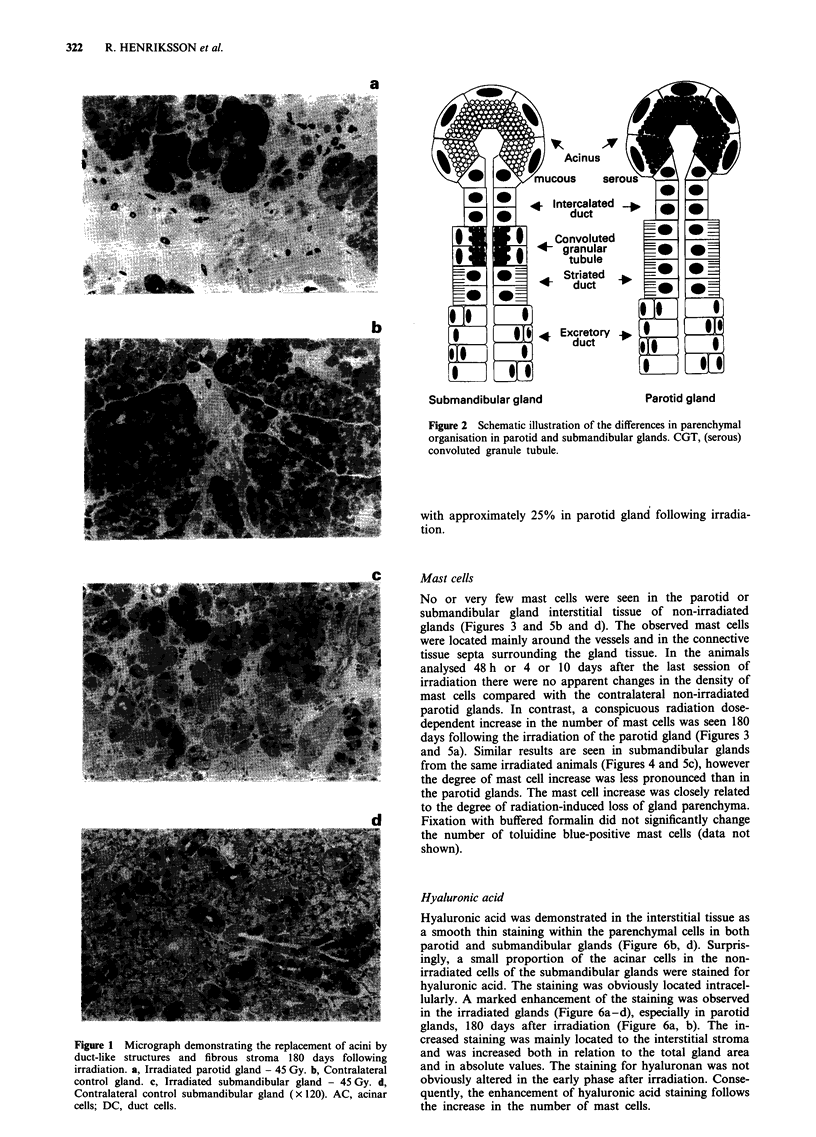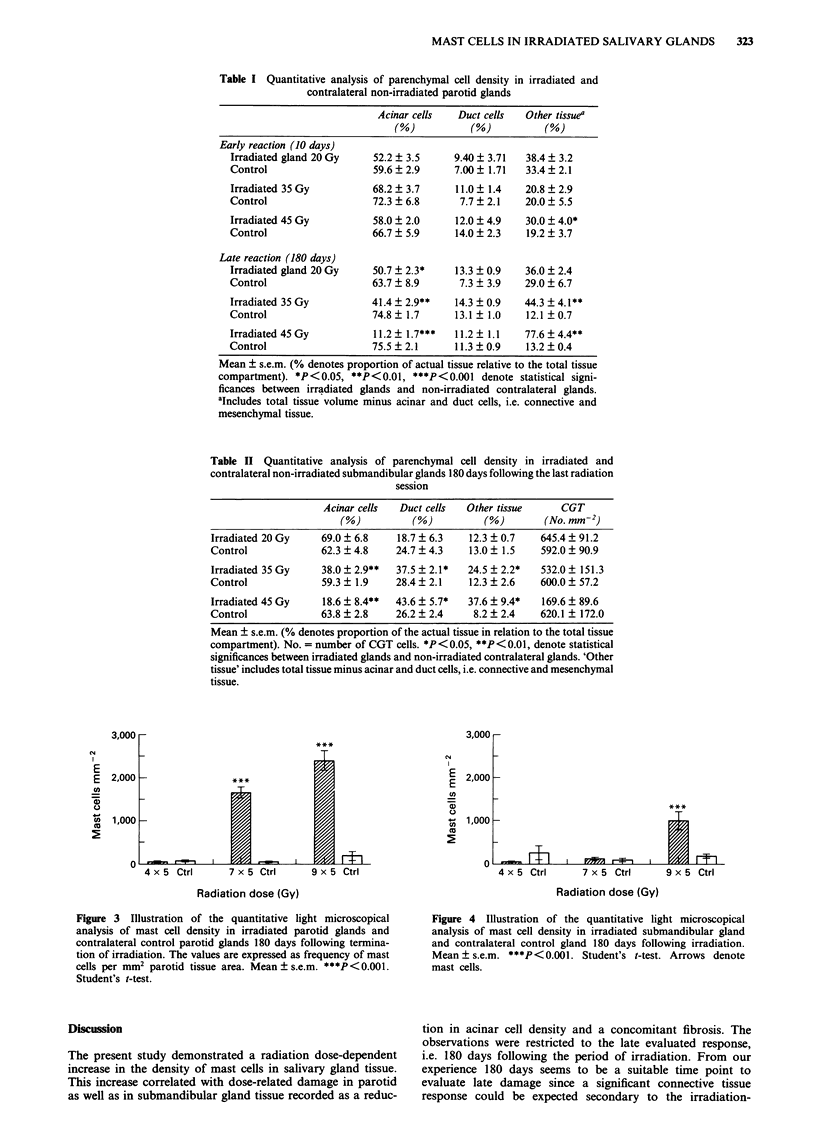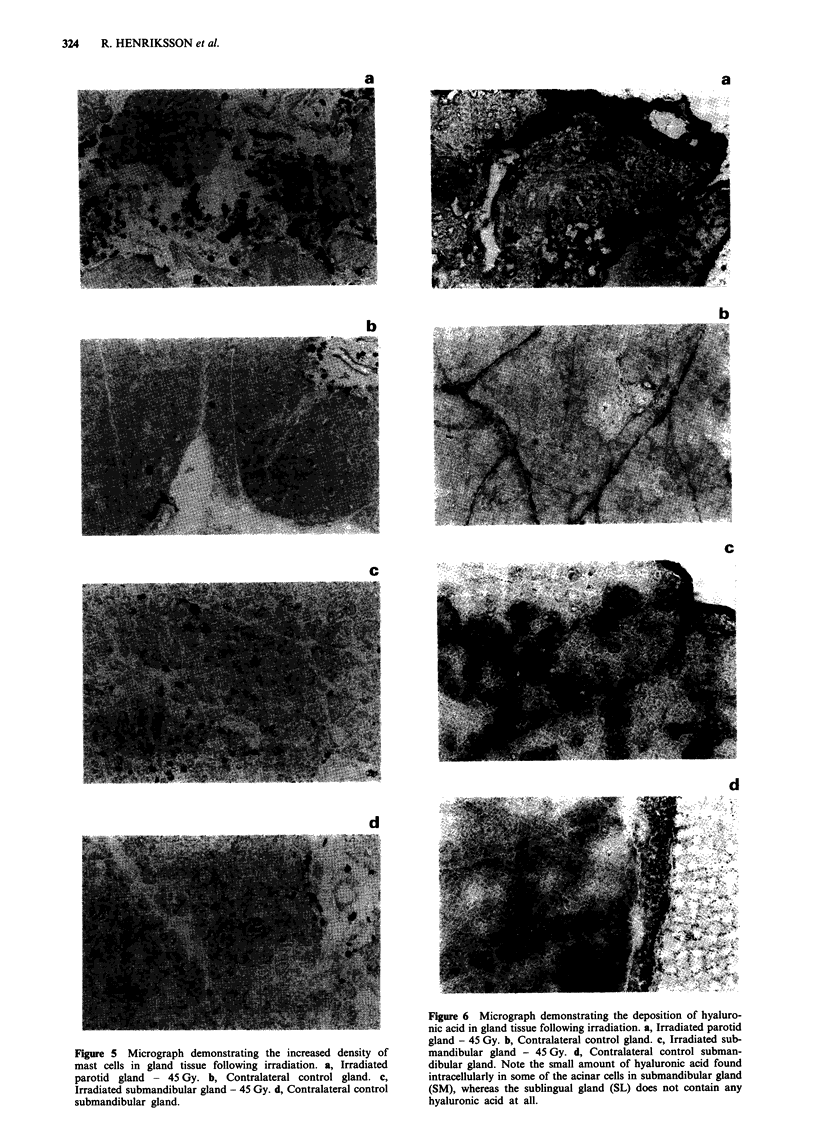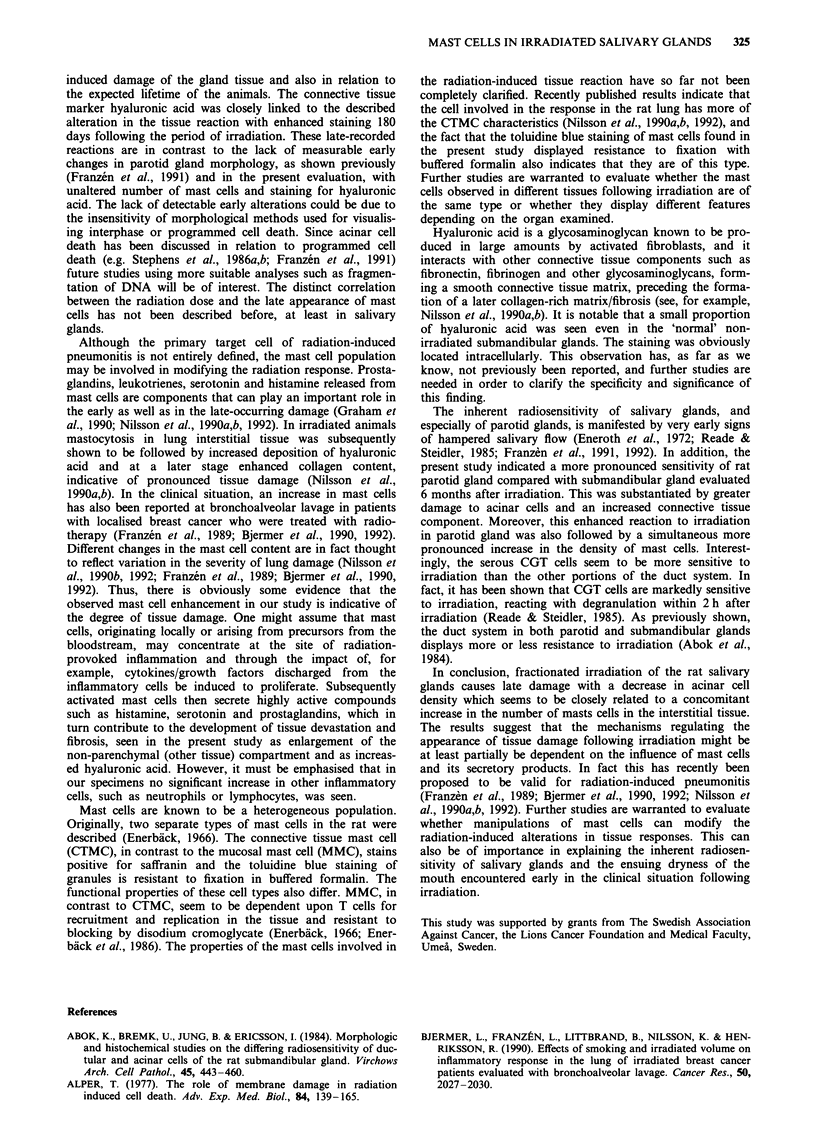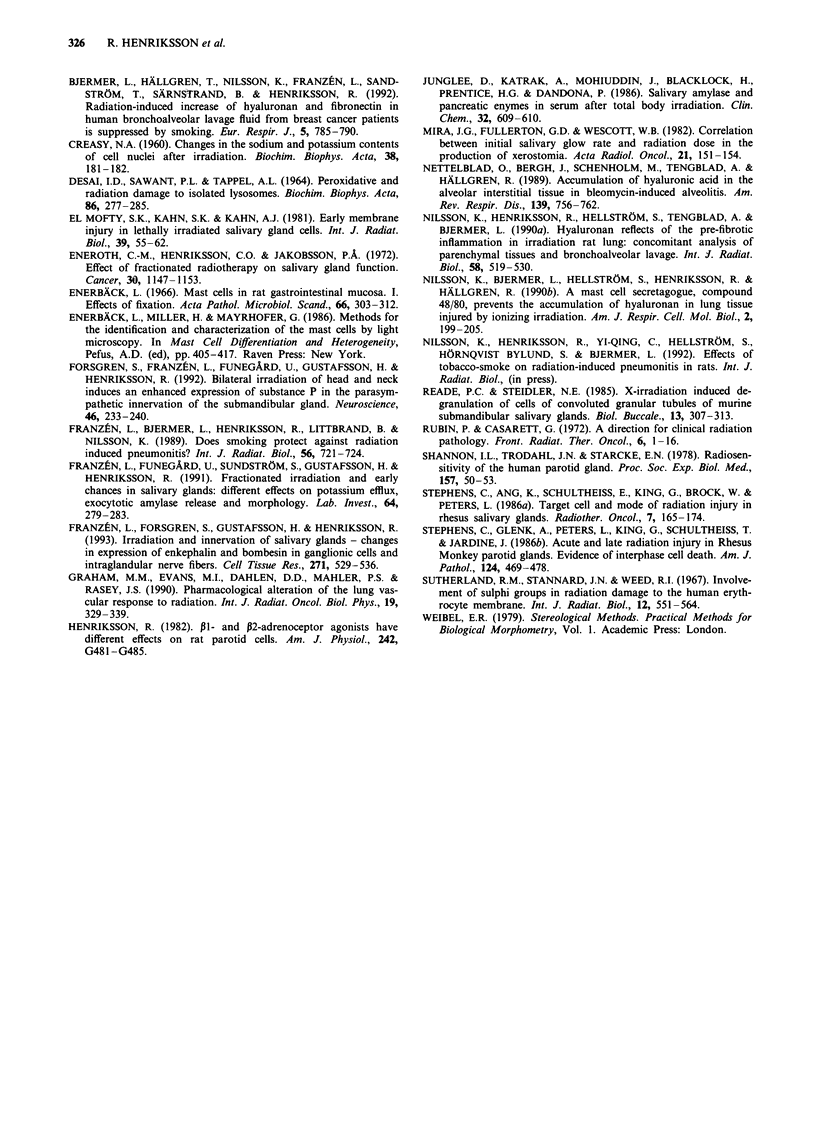# Increase in mast cells and hyaluronic acid correlates to radiation-induced damage and loss of serous acinar cells in salivary glands: the parotid and submandibular glands differ in radiation sensitivity.

**DOI:** 10.1038/bjc.1994.58

**Published:** 1994-02

**Authors:** R. Henriksson, O. Fröjd, H. Gustafsson, S. Johansson, C. Yi-Qing, L. Franzén, L. Bjermer

**Affiliations:** Department of Oncology, University Hospital, Umeå, Sweden.

## Abstract

**Images:**


*Br. J. Cancer* (1994), **69**, 320–326 © Macmillan Press Ltd., 1994

### Increase in mast cells and hyaluronic acid correlates to radiation-induced damage and loss of serous acinar cells in salivary glands: the parotid and submandibular glands differ in radiation sensitivity

R. Henriksson[1], Ö. Fröjd[1], H. Gustafsson[2], S. Johansson[1], C. Yi-Qing[1], L. Franzén[1] & L. Bjermer[3]

*Departments of [1]Oncology and [2]Oto-rhino-laryngology, University Hospital, S-901 87 Umeå, Sweden; [3]Department of Lung Medicine, University Hospital, Trondheim, Norway*

**Summary** The detailed mechanisms which can explain the inherent radiosensitivity of salivary glands remain to be elucidated. Although DNA is the most plausible critical target for the lethal effects of irradiation, interactions with other constituents, such as cell membrane and neuropeptides, have been suggested to cause important physiological changes. Moreover, mast cells seem to be closely linked to radiation-induced pneumonitis. Therefore, in the present study the effects of fractionated irradiation on salivary glands have been assessed with special regard to the appearance of mast cells and its correlation with damage to gland parenchyma. Sprague–Dawley strain rats were unilaterally irradiated to the head and neck with the salivary glands within the radiation field. The irradiation was delivered once daily for 5 days to a total dose of 20, 35 and 45 Gy. The contralateral parotid and submandibular glands served as intra-animal controls and parallel analysis of glands was performed 2, 4, 10 or 180 days following the last radiation treatment. Morphological analysis revealed no obvious changes up to 10 days after the irradiation. At 180 days a radiation dose-dependent loss of gland parenchyma was seen, especially with regard to serous acinar cells in parotid gland and acinar cells and serous CGT (convoluted granular tubule) cells in the submandibular gland. These changes displayed a close correlation with a concomitant dose-dependent enhanced density of mast cells and staining for hyaluronic acid. This cell population seems to conform with the features of the connective tissue mast cell type. The parotid seems to be more sensitive to irradiation than the submandibular gland. Thus, the present results further strengthen the role of and the potential interaction of mast cells with radiation-induced tissue injury and alterations in normal tissue integrity.

Although the detailed mechanisms by which radiation induces damage in biological tissues are not fully understood, it is generally accepted that generation of free radicals is of major importance. DNA is the most likely target for the lethal effects of these irradiation induced free radicals, however it is plausible that other compartments, i.e. non-genetic macromolecules, such as proteins and carbohydrates, can be significantly affected with vital alterations in the functional integrity of the certain biological tissues (Creasy, 1960; Desai *et al.*, 1964; Sutherland *et al.*, 1967; Alper, 1977; El-Mofty *et al.*, 1981; Franzén *et al.*, 1991; 1993). In addition, changes caused by external factors and in the surrounding tissues can play an important role in modifying the final detectable and evaluable reaction, which indeed has recently been proposed, e.g. ongoing smoking depressed pneumonitis caused by irradiation in humans (Franzén *et al.*, 1989; Bjermer *et al.*, 1990; 1992) as well as in experimental animals (Nilsson *et al.*, 1992). Moreover, sublethal damage following fractionated irradiation seems to cause alterations in cell membrane function and in physiological parameters (Reade & Steidler, 1985; Stephens *et al.*, 1986*a*,*b*; Franzén *et al.*, 1991). Most recently, it was also demonstrated that irradiation enhances the expression of important neutopeptides/growth factors, at least in salivary glands (Forsgren *et al.*, 1992; Franzén *et al.*, 1993). Furthermore, parotid acinar cell death following irradiation has been described as an interphase death (Stephens *et al.*, 1986*a*,*b*; Franzén *et al.*, 1991).

The remarkable sensitivity of salivary gland acinar cells compared with other glands to radiation is a unique radiobiological phenomenon (Shannon *et al.*, 1978; Mira *et al.*, 1982; Abok *et al.*, 1984; Junglee *et al.*, 1986; Franzén *et al.*, 1991). Other well-differentiated gland cells are known to be more or less radioresistant (Rubin & Casarett, 1972). The exact mechanism which explains this sensitivity is not entirely understood. Since mast cells have been proposed to interact in a substantial manner with the development of radiation-induced pneumonitis and pulmonary fibrosis (Franzén *et al.*, 1989; Bjermer *et al.*, 1990; Nilsson *et al.*, 1990*a*,*b*; 1992) we found it of interest to evaluate the correlation of connective tissue response and especially mast cells with radiation-induced damage in salivary gland tissue.

Correspondence: R. Henriksson.
Received 2 February 1993; and in revised form 24 June 1993.

### Materials and methods

### *Animals*

Fifty white albino female rats of the Sprague–Dawley strain were used. They were 8 weeks old, and weighed approximately 200 g. They were fed water and chow *ad libitum* and kept on a diurnal light schedule. Before the animals were used for the experiments outlined below they were fasted for 18 h, and used before noon to avoid diurnal variations.

### *Irradiation procedure*

The rats were irradiated with a medical linear accelerator (BBC[R] 6 MV with a dose rate of 2.19 Gy min$^{-1}$; at a focus to skin distance of 100 cm). The rats under methohexital (Brietal) anaesthesia were placed in a plastic mould holding them firmly in position during irradiation. During this time the rats were observed with a TV camera and if they moved the treatment was temporarily interrupted. The total radiation field used on the accelerator was 8 × 20 cm, allowing two rats to be irradiated at the same time. One side of the head (the reference side) was not irradiated; it was also shielded with a 10-cm-thick lead block. By this technique, the non-irradiated side was effectively protected. The geometric margins between the edge of the radiation field and parotid glands were 10 mm in all cases, and the distance between the field edge and the 95% dose level of this beam was 6–7 mm. According to this the minumum dose to the salivary glands on the irradiated side was at least 95% of the prescribed dose. The dose contribution on the reference side was insignificant. The absolute dosimetry was checked with an ionisation chamber in a rat phantom and all scattering materials in the field were kept constant. The animals were irradiated daily Monday to Friday with daily doses of 4, 7 or 9 Gy up to a total dose of 20, 35 or 45 Gy respectively. The animals were used for the experimental procedure as outlined below 48 h and 4, 10 and 180 days following the last irradiation treatment. These endpoint times were chosen to detect the appearance of early and late effects because the rodents have a short life expectancy. However, it must be emphasised that we have no conclusive results regarding the optimal time point for examining early or late effects.

### *Morphological evaluations*

The parotid and submandibular gland specimens from five animals at each time point and dose were fixed in 3% glutaraldehyde in 0.2 M phosphate buffer immediately after removal. After rinsing in buffer, the salivary gland specimens were post fixed in 1% osmium tetroxide in the same buffer. After a cold buffer rinse, the specimens were dehydrated in graded ethanol solutions and embedded in epoxy resin. Semi-thin (1 μm) sections, cut on an LKB ultratome and stained with toluidine blue, were used for light microscopic analysis and morphometry. The latter was performed by using an ocular with 10 × 10 grid mesh and counting 'hits' at the crossings (cross-points). One hundred points per section were counted (Weibel, 1979; Henriksson, 1982). Specimens from both irradiated glands and contralateral controls were fixed and embedded in parallel (Henriksson, 1982; Franzén *et al.*, 1991). In the submandibular gland CGT (convoluted granule tubule) serous cells in the ducts were counted with two ocular fields (limited by the outer border of the mesh grid) to a total 0.125 $mm^2$ in each gland. The results were then calculated and are expressed as number of CGT cells per $mm^2$ gland area.

*Mast cell analysis* Cryosections 5 μm thick were stained with 0.5% toluidine blue in hydrochloric acid (0.5 M) and counterstained with Mayer's acid haematoxylin. A total gland area of 0.452 $mm^2$ was scanned through a microscope at 240 × magnification and the numbers of toluidine blue-positive mast cells were counted and presented as the total number of mast cells per $mm^2$ for each gland. Parallel sections were fixed in 4% buffered formaldehyde overnight and then stained with toluidine blue (Nilsson *et al.*, 1990*a,b*). Randomly selected areas were compared regarding numbers of mast cells.

*Localisation of hyaluronan (hyaluronic acid) by light microscopy* In brief, cryostatic sections from the salivary glands were cut with a microtome into 1.5-μm-thick sections and incubated for 30 min with 1% bovine serum albumin in PBS. The sections were then incubated with a hyaluronan binding probe (biotin-labelled HABP, 85 $\mu g\ ml^{-1}$, Pharmacia Diagnostics, Uppsala, Sweden) overnight at 4°C and washed with PBS twice for 20 min before being treated with avidin-biotin-peroxidase (Vectastain, Vector Lab, Burlingam, CA, USA, 1:200 dilution) for 1 h (see Nettelblad *et al.*, 1989; Nilsson *et al.*, 1990). After three washes in PBS, the sections were incubated for 5 min in 0.05% diaminobenzidine (Sigma, USA) and 0.03% hydrogen peroxidase in phosphate buffer at room temperature. Some sections were incubated with *Streptomyces* hyaluronidase (Sigma, USA) in the presence of the protease inhibitors in PBS for 3 h at 37°C, and washed with PBS twice for 10 min prior to staining. The digestion experiments included controls incubated under otherwise similar conditions but without the enzyme (Nettelblad *et al.*, 1989; Nilsson *et al.*, 1990*a*; 1992).

### *Statistical evaluation*

All statistical analysis were performed on an IBM PS/2 and using CSS/statistical software (Statsoft, Tulsa, OK, USA). Student's *t*-test was used for comparisons between groups.

### Results

The acute effects of the radiation schedule used in these studies on the general condition of the rats have been described in previous papers (Franzén *et al.*, 1991). Only the animals that received the highest doses displayed a marked erythemal reaction corresponding to the radiation field, approximately 10–14 days following irradiation. In the same rats oral mucositis was also observed. The animals were supplied with subcutaneous injections of physiological saline during the first 10 days. However, the mucositis did not during the observed period (limited to 180 days) influence to any substantial degree the animals' ability to eat. Consequently, there were no significant differences in weight gain between irradiated animals and controls during the follow-up period. At the late time point (180 days) the animals irradiated with 45 Gy displayed a lower density of body hair in the irradiation field.

### *General morphology*

*Parotid vs submandibular gland* On the non-irradiated side the well-known lobular structure, with densely packed acini and ducts, was seen in all specimens (Figure 1b). The parotid gland consists solely of serous acinar cells with branching intercalated ducts between the acini. The gland is clearly lobulated with easily distinguished acini and ducts. Myoepithelial cells are seen surrounding the acini and the intercalated ducts. Striated ducts and the interlobular excretory ducts are readily differentiated from acini. The interlobular stroma is more apparent than in submandibular gland. The submandibular gland in the rat (Figure 1d), on the other hand, includes only mucous acinar cells with few myoepithelial cells. Convoluted granular tubules (CGT) incorporating intensive toluidine blue-stained serous cells make up roughly 10% of section area, and striated duct cells with a tall cylindrical shape and centrally positioned nuclei make up a similar proportion of the tissue. The interlobular stroma is very scant and, besides excretory ducts, arteries and veins, only isolated mesenchymal cells are observed. The principal differences between parotid and submandibular glands in the rat are outlined schematically in Figure 2.

*Early effects* Irradiation caused no significant quantitative or qualitative changes in the parotid gland tissues by 10 days after the last session of irradiation (Table I). The structure of irradiated and control acinar cells was in accordance with earlier analysis (e.g. Henriksson, 1982). No obvious changes were seen in the irradiated submandibular gland compared with control glands. No changes in acinar cells or the occurrence of mast cells were observed 2 or 4 days after initiation of the irradiation.

*Late effects* At 180 days following the irradiation a marked alteration in the parotid gland morphology was observed (Figure 1a). This was especially marked in the animals treated with higher doses, in which a huge loss of acinar cells was evident (Table I). The quantitative measurements of the various parenchymal cells demonstrated a dose-dependent decline in the density of acinar cells, whereas a concomitant increase in connective tissue was evident. Signs of oedema formation were also evident. The duct cells were seemingly preserved. Otherwise, the morphology of parotid acinar cells of irradiated and control glands was similar to that reported in previous investigations (Henriksson, 1982; Franzén *et al.*, 1991) with an intact basement membrane. In submandibular glands (Figure 1c) a similar loss of secretory cells was seen following irradiation (Table II). The decrease in mucous acinar cells, however, was not as pronounced as that of the serous cells in parotid gland. The serous CGT cells in the duct system were significantly affected by the irradiation, especially at the highest radiation dose (Table II). In contrast to the parotid gland there was a marked relative increase in the duct cells. In total the parenchymal cells (acini + ducts) proportion was 60% in submandibular gland as compared

**Figure 1** Micrograph demonstrating the replacement of acini by duct-like structures and fibrous stroma 180 days following irradiation. **a**, Irradiated parotid gland – 45 Gy. **b**, Contralateral control gland. **c**, Irradiated submandibular gland – 45 Gy. **d**, Contralateral control submandibular gland (×120). AC, acinar cells; DC, duct cells.

**Figure 2** Schematic illustration of the differences in parenchymal organisation in parotid and submandibular glands. CGT, (serous) convoluted granule tubule.

with approximately 25% in parotid gland following irradiation.

*Mast cells*

No or very few mast cells were seen in the parotid or submandibular gland interstitial tissue of non-irradiated glands (Figures 3 and 5b and d). The observed mast cells were located mainly around the vessels and in the connective tissue septa surrounding the gland tissue. In the animals analysed 48 h or 4 or 10 days after the last session of irradiation there were no apparent changes in the density of mast cells compared with the contralateral non-irradiated parotid glands. In contrast, a conspicuous radiation dose-dependent increase in the number of mast cells was seen 180 days following the irradiation of the parotid gland (Figures 3 and 5a). Similar results are seen in submandibular glands from the same irradiated animals (Figures 4 and 5c), however the degree of mast cell increase was less pronounced than in the parotid glands. The mast cell increase was closely related to the degree of radiation-induced loss of gland parenchyma. Fixation with buffered formalin did not significantly change the number of toluidine blue-positive mast cells (data not shown).

*Hyaluronic acid*

Hyaluronic acid was demonstrated in the interstitial tissue as a smooth thin staining within the parenchymal cells in both parotid and submandibular glands (Figure 6b, d). Surprisingly, a small proportion of the acinar cells in the non-irradiated cells of the submandibular glands were stained for hyaluronic acid. The staining was obviously located intracellularly. A marked enhancement of the staining was observed in the irradiated glands (Figure 6a–d), especially in parotid glands, 180 days after irradiation (Figure 6a, b). The increased staining was mainly located to the interstitial stroma and was increased both in relation to the total gland area and in absolute values. The staining for hyaluronan was not obviously altered in the early phase after irradiation. Consequently, the enhancement of hyaluronic acid staining follows the increase in the number of mast cells.

**Table I** Quantitative analysis of parenchymal cell density in irradiated and contralateral non-irradiated parotid glands

| | *Acinar cells (%)* | *Duct cells (%)* | *Other tissue[a] (%)* |
|---|---|---|---|
| *Early reaction (10 days)* | | | |
| Irradiated gland 20 Gy | 52.2 ± 3.5 | 9.40 ± 3.71 | 38.4 ± 3.2 |
| Control | 59.6 ± 2.9 | 7.00 ± 1.71 | 33.4 ± 2.1 |
| Irradiated 35 Gy | 68.2 ± 3.7 | 11.0 ± 1.4 | 20.8 ± 2.9 |
| Control | 72.3 ± 6.8 | 7.7 ± 2.1 | 20.0 ± 5.5 |
| Irradiated 45 Gy | 58.0 ± 2.0 | 12.0 ± 4.9 | 30.0 ± 4.0* |
| Control | 66.7 ± 5.9 | 14.0 ± 2.3 | 19.2 ± 3.7 |
| *Late reaction (180 days)* | | | |
| Irradiated gland 20 Gy | 50.7 ± 2.3* | 13.3 ± 0.9 | 36.0 ± 2.4 |
| Control | 63.7 ± 8.9 | 7.3 ± 3.9 | 29.0 ± 6.7 |
| Irradiated 35 Gy | 41.4 ± 2.9** | 14.3 ± 0.9 | 44.3 ± 4.1** |
| Control | 74.8 ± 1.7 | 13.1 ± 1.0 | 12.1 ± 0.7 |
| Irradiated 45 Gy | 11.2 ± 1.7*** | 11.2 ± 1.1 | 77.6 ± 4.4** |
| Control | 75.5 ± 2.1 | 11.3 ± 0.9 | 13.2 ± 0.4 |

Mean ± s.e.m. (% denotes proportion of actual tissue relative to the total tissue compartment). *$P<0.05$, **$P<0.01$, ***$P<0.001$ denote statistical significances between irradiated glands and non-irradiated contralateral glands. [a]Includes total tissue volume minus acinar and duct cells, i.e. connective and mesenchymal tissue.

**Table II** Quantitative analysis of parenchymal cell density in irradiated and contralateral non-irradiated submandibular glands 180 days following the last radiation session

| | *Acinar cells (%)* | *Duct cells (%)* | *Other tissue (%)* | *CGT (No. mm$^{-2}$)* |
|---|---|---|---|---|
| Irradiated 20 Gy | 69.0 ± 6.8 | 18.7 ± 6.3 | 12.3 ± 0.7 | 645.4 ± 91.2 |
| Control | 62.3 ± 4.8 | 24.7 ± 4.3 | 13.0 ± 1.5 | 592.0 ± 90.9 |
| Irradiated 35 Gy | 38.0 ± 2.9** | 37.5 ± 2.1* | 24.5 ± 2.2* | 532.0 ± 151.3 |
| Control | 59.3 ± 1.9 | 28.4 ± 2.1 | 12.3 ± 2.6 | 600.0 ± 57.2 |
| Irradiated 45 Gy | 18.6 ± 8.4** | 43.6 ± 5.7* | 37.6 ± 9.4* | 169.6 ± 89.6 |
| Control | 63.8 ± 2.8 | 26.2 ± 2.4 | 8.2 ± 2.4 | 620.1 ± 172.0 |

Mean ± s.e.m. (% denotes proportion of the actual tissue in relation to the total tissue compartment). No. = number of CGT cells. *$P<0.05$, **$P<0.01$, denote statistical significances between irradiated glands and non-irradiated contralateral glands. 'Other tissue' includes total tissue minus acinar and duct cells, i.e. connective and mesenchymal tissue.

**Figure 3** Illustration of the quantitative light microscopical analysis of mast cell density in irradiated parotid glands and contralateral control parotid glands 180 days following termination of irradiation. The values are expressed as frequency of mast cells per mm$^2$ parotid tissue area. Mean ± s.e.m. ***$P<0.001$. Student's *t*-test.

**Figure 4** Illustration of the quantitative light microscopical analysis of mast cell density in irradiated submandibular gland and contralateral control gland 180 days following irradiation. Mean ± s.e.m. ***$P<0.001$. Student's *t*-test. Arrows denote mast cells.

### Discussion

The present study demonstrated a radiation dose-dependent increase in the density of mast cells in salivary gland tissue. This increase correlated with dose-related damage in parotid as well as in submandibular gland tissue recorded as a reduction in acinar cell density and a concomitant fibrosis. The observations were restricted to the late evaluated response, i.e. 180 days following the period of irradiation. From our experience 180 days seems to be a suitable time point to evaluate late damage since a significant connective tissue response could be expected secondary to the irradiation-

**Figure 5** Micrograph demonstrating the increased density of mast cells in gland tissue following irradiation. **a**, Irradiated parotid gland – 45 Gy. **b**, Contralateral control gland. **c**, Irradiated submandibular gland – 45 Gy. **d**, Contralateral control submandibular gland.

**Figure 6** Micrograph demonstrating the deposition of hyaluronic acid in gland tissue following irradiation. **a**, Irradiated parotid gland – 45 Gy. **b**, Contralateral control gland. **c**, Irradiated submandibular gland – 45 Gy. **d**, Contralateral control submandibular gland. Note the small amount of hyaluronic acid found intracellularly in some of the acinar cells in submandibular gland (SM), whereas the sublingual gland (SL) does not contain any hyaluronic acid at all.

induced damage of the gland tissue and also in relation to the expected lifetime of the animals. The connective tissue marker hyaluronic acid was closely linked to the described alteration in the tissue reaction with enhanced staining 180 days following the period of irradiation. These late-recorded reactions are in contrast to the lack of measurable early changes in parotid gland morphology, as shown previously (Franzén *et al.*, 1991) and in the present evaluation, with unaltered number of mast cells and staining for hyaluronic acid. The lack of detectable early alterations could be due to the insensitivity of morphological methods used for visualising interphase or programmed cell death. Since acinar cell death has been discussed in relation to programmed cell death (e.g. Stephens *et al.*, 1986*a*,*b*; Franzén *et al.*, 1991) future studies using more suitable analyses such as fragmentation of DNA will be of interest. The distinct correlation between the radiation dose and the late appearance of mast cells has not been described before, at least in salivary glands.

Although the primary target cell of radiation-induced pneumonitis is not entirely defined, the mast cell population may be involved in modifying the radiation response. Prostaglandins, leukotrienes, serotonin and histamine released from mast cells are components that can play an important role in the early as well as in the late-occurring damage (Graham *et al.*, 1990; Nilsson *et al.*, 1990*a*,*b*, 1992). In irradiated animals mastocytosis in lung interstitial tissue was subsequently shown to be followed by increased deposition of hyaluronic acid and at a later stage enhanced collagen content, indicative of pronounced tissue damage (Nilsson *et al.*, 1990*a*,*b*). In the clinical situation, an increase in mast cells has also been reported at bronchoalveolar lavage in patients with localised breast cancer who were treated with radiotherapy (Franzén *et al.*, 1989; Bjermer *et al.*, 1990, 1992). Different changes in the mast cell content are in fact thought to reflect variation in the severity of lung damage (Nilsson *et al.*, 1990*b*, 1992; Franzén *et al.*, 1989; Bjermer *et al.*, 1990, 1992). Thus, there is obviously some evidence that the observed mast cell enhancement in our study is indicative of the degree of tissue damage. One might assume that mast cells, originating locally or arising from precursors from the bloodstream, may concentrate at the site of radiation-provoked inflammation and through the impact of, for example, cytokines/growth factors discharged from the inflammatory cells be induced to proliferate. Subsequently activated mast cells then secrete highly active compounds such as histamine, serotonin and prostaglandins, which in turn contribute to the development of tissue devastation and fibrosis, seen in the present study as enlargement of the non-parenchymal (other tissue) compartment and as increased hyaluronic acid. However, it must be emphasised that in our specimens no significant increase in other inflammatory cells, such as neutrophils or lymphocytes, was seen.

Mast cells are known to be a heterogeneous population. Originally, two separate types of mast cells in the rat were described (Enerbäck, 1966). The connective tissue mast cell (CTMC), in contrast to the mucosal mast cell (MMC), stains positive for saffranin and the toluidine blue staining of granules is resistant to fixation in buffered formalin. The functional properties of these cell types also differ. MMC, in contrast to CTMC, seem to be dependent upon T cells for recruitment and replication in the tissue and resistant to blocking by disodium cromoglycate (Enerbäck, 1966; Enerbäck *et al.*, 1986). The properties of the mast cells involved in the radiation-induced tissue reaction have so far not been completely clarified. Recently published results indicate that the cell involved in the response in the rat lung has more of the CTMC characteristics (Nilsson *et al.*, 1990*a*,*b*, 1992), and the fact that the toluidine blue staining of mast cells found in the present study displayed resistance to fixation with buffered formalin also indicates that they are of this type. Further studies are warranted to evaluate whether the mast cells observed in different tissues following irradiation are of the same type or whether they display different features depending on the organ examined.

Hyaluronic acid is a glycosaminoglycan known to be produced in large amounts by activated fibroblasts, and it interacts with other connective tissue components such as fibronectin, fibrinogen and other glycosaminoglycans, forming a smooth connective tissue matrix, preceding the formation of a later collagen-rich matrix/fibrosis (see, for example, Nilsson *et al.*, 1990*a*,*b*). It is notable that a small proportion of hyaluronic acid was seen even in the 'normal' non-irradiated submandibular glands. The staining was obviously located intracellularly. This observation has, as far as we know, not previously been reported, and further studies are needed in order to clarify the specificity and significance of this finding.

The inherent radiosensitivity of salivary glands, and especially of parotid glands, is manifested by very early signs of hampered salivary flow (Eneroth *et al.*, 1972; Reade & Steidler, 1985; Franzèn *et al.*, 1991, 1992). In addition, the present study indicated a more pronounced sensitivity of rat parotid gland compared with submandibular gland evaluated 6 months after irradiation. This was substantiated by greater damage to acinar cells and an increased connective tissue component. Moreover, this enhanced reaction to irradiation in parotid gland was also followed by a simultaneous more pronounced increase in the density of mast cells. Interestingly, the serous CGT cells seem to be more sensitive to irradiation than the other portions of the duct system. In fact, it has been shown that CGT cells are markedly sensitive to irradiation, reacting with degranulation within 2 h after irradiation (Reade & Steidler, 1985). As previously shown, the duct system in both parotid and submandibular glands displays more or less resistance to irradiation (Abok *et al.*, 1984).

In conclusion, fractionated irradiation of the rat salivary glands causes late damage with a decrease in acinar cell density which seems to be closely related to a concomitant increase in the number of masts cells in the interstitial tissue. The results suggest that the mechanisms regulating the appearance of tissue damage following irradiation might be at least partially be dependent on the influence of mast cells and its secretory products. In fact this has recently been proposed to be valid for radiation-induced pneumonitis (Franzèn *et al.*, 1989; Bjermer *et al.*, 1990, 1992; Nilsson *et al.*, 1990*a*,*b*, 1992). Further studies are warranted to evaluate whether manipulations of mast cells can modify the radiation-induced alterations in tissue responses. This can also be of importance in explaining the inherent radiosensitivity of salivary glands and the ensuing dryness of the mouth encountered early in the clinical situation following irradiation.

This study was supported by grants from The Swedish Association Against Cancer, the Lions Cancer Foundation and Medical Faculty, Umeå, Sweden.

### References

ABOK, K., BREMK, U., JUNG, B. & ERICSSON, I. (1984). Morphologic and histochemical studies on the differing radiosensitivity of ductular and acinar cells of the rat submandibular gland. *Virchows Arch. Cell Pathol.*, **45**, 443–460.

ALPER, T. (1977). The role of membrane damage in radiation induced cell death. *Adv. Exp. Med. Biol.*, **84**, 139–165.

BJERMER, L., FRANZÉN, L., LITTBRAND, B., NILSSON, K. & HENRIKSSON, R. (1990). Effects of smoking and irradiated volume on inflammatory response in the lung of irradiated breast cancer patients evaluated with bronchoalveolar lavage. *Cancer Res.*, **50**, 2027–2030.

BJERMER, L., HÄLLGREN, T., NILSSON, K., FRANZÉN, L., SANDSTRÖM, T., SÄRNSTRAND, B. & HENRIKSSON, R. (1992). Radiation-induced increase of hyaluronan and fibronectin in human bronchoalveolar lavage fluid from breast cancer patients is suppressed by smoking. *Eur. Respir. J.*, **5**, 785–790.

CREASY, N.A. (1960). Changes in the sodium and potassium contents of cell nuclei after irradiation. *Biochim. Biophys. Acta*, **38**, 181–182.

DESAI, I.D., SAWANT, P.L. & TAPPEL, A.L. (1964). Peroxidative and radiation damage to isolated lysosomes. *Biochim. Biophys. Acta*, **86**, 277–285.

EL MOFTY, S.K., KAHN, S.K. & KAHN, A.J. (1981). Early membrane injury in lethally irradiated salivary gland cells. *Int. J. Radiat. Biol.*, **39**, 55–62.

ENEROTH, C.-M., HENRIKSSON, C.O. & JAKOBSSON, P.Å. (1972). Effect of fractionated radiotherapy on salivary gland function. *Cancer*, **30**, 1147–1153.

ENERBÄCK, L. (1966). Mast cells in rat gastrointestinal mucosa. I. Effects of fixation. *Acta Pathol. Microbiol. Scand.*, **66**, 303–312.

ENERBÄCK, L., MILLER, H. & MAYRHOFER, G. (1986). Methods for the identification and characterization of the mast cells by light microscopy. In *Mast Cell Differentiation and Heterogeneity*, Pefus, A.D. (ed), pp. 405–417. Raven Press: New York.

FORSGREN, S., FRANZÉN, L., FUNEGÅRD, U., GUSTAFSSON, H. & HENRIKSSON, R. (1992). Bilateral irradiation of head and neck induces an enhanced expression of substance P in the parasympathetic innervation of the submandibular gland. *Neuroscience*, **46**, 233–240.

FRANZÉN, L., BJERMER, L., HENRIKSSON, R., LITTBRAND, B. & NILSSON, K. (1989). Does smoking protect against radiation induced pneumonitis? *Int. J. Radiat. Biol.*, **56**, 721–724.

FRANZÉN, L., FUNEGÅRD, U., SUNDSTRÖM, S., GUSTAFSSON, H. & HENRIKSSON, R. (1991). Fractionated irradiation and early chances in salivary glands: different effects on potassium efflux, exocytotic amylase release and morphology. *Lab. Invest.*, **64**, 279–283.

FRANZÉN, L., FORSGREN, S., GUSTAFSSON, H. & HENRIKSSON, R. (1993). Irradiation and innervation of salivary glands – changes in expression of enkephalin and bombesin in ganglionic cells and intraglandular nerve fibers. *Cell Tissue Res.*, **271**, 529–536.

GRAHAM, M.M., EVANS, M.I., DAHLEN, D.D., MAHLER, P.S. & RASEY, J.S. (1990). Pharmacological alteration of the lung vascular response to radiation. *Int. J. Radiat. Oncol. Biol. Phys.*, **19**, 329–339.

HENRIKSSON, R. (1982). $\beta$1- and $\beta$2-adrenoceptor agonists have different effects on rat parotid cells. *Am. J. Physiol.*, **242**, G481–G485.

JUNGLEE, D., KATRAK, A., MOHIUDDIN, J., BLACKLOCK, H., PRENTICE, H.G. & DANDONA, P. (1986). Salivary amylase and pancreatic enymes in serum after total body irradiation. *Clin. Chem.*, **32**, 609–610.

MIRA, J.G., FULLERTON, G.D. & WESCOTT, W.B. (1982). Correlation between initial salivary glow rate and radiation dose in the production of xerostomia. *Acta Radiol. Oncol.*, **21**, 151–154.

NETTELBLAD, O., BERGH, J., SCHENHOLM, M., TENGBLAD, A. & HÄLLGREN, R. (1989). Accumulation of hyaluronic acid in the alveolar interstitial tissue in bleomycin-induced alveolitis. *Am. Rev. Respir. Dis.*, **139**, 756–762.

NILSSON, K., HENRIKSSON, R., HELLSTRÖM, S., TENGBLAD, A. & BJERMER, L. (1990*a*). Hyaluronan reflects of the pre-fibrotic inflammation in irradiation rat lung: concomitant analysis of parenchymal tissues and bronchoalveolar lavage. *Int. J. Radiat. Biol.*, **58**, 519–530.

NILSSON, K., BJERMER, L., HELLSTRÖM, S., HENRIKSSON, R. & HÄLLGREN, R. (1990*b*). A mast cell secretagogue, compound 48/80, prevents the accumulation of hyaluronan in lung tissue injured by ionizing irradiation. *Am. J. Respir. Cell. Mol. Biol.*, **2**, 199–205.

NILSSON, K., HENRIKSSON, R., YI-QING, C., HELLSTRÖM, S., HÖRNQVIST BYLUND, S. & BJERMER, L. (1992). Effects of tobacco-smoke on radiation-induced pneumonitis in rats. *Int. J. Radiat. Biol.*, (in press).

READE, P.C. & STEIDLER, N.E. (1985). X-irradiation induced degranulation of cells of convoluted granular tubules of murine submandibular salivary glands. *Biol. Buccale.*, **13**, 307–313.

RUBIN, P. & CASARETT, G. (1972). A direction for clinical radiation pathology. *Front. Radiat. Ther. Oncol.*, **6**, 1–16.

SHANNON, I.L., TRODAHL, J.N. & STARCKE, E.N. (1978). Radiosensitivity of the human parotid gland. *Proc. Soc. Exp. Biol. Med.*, **157**, 50–53.

STEPHENS, C., ANG, K., SCHULTHEISS, E., KING, G., BROCK, W. & PETERS, L. (1986*a*). Target cell and mode of radiation injury in rhesus salivary glands. *Radiother. Oncol.*, **7**, 165–174.

STEPHENS, C., GLENK, A., PETERS, L., KING, G., SCHULTHEISS, T. & JARDINE, J. (1986*b*). Acute and late radiation injury in Rhesus Monkey parotid glands. Evidence of interphase cell death. *Am. J. Pathol.*, **124**, 469–478.

SUTHERLAND, R.M., STANNARD, J.N. & WEED, R.I. (1967). Involvement of sulphi groups in radiation damage to the human erythrocyte membrane. *Int. J. Radiat. Biol.*, **12**, 551–564.

WEIBEL, E.R. (1979). *Stereological Methods. Practical Methods for Biological Morphometry*, Vol. 1. Academic Press: London.